# Novel Duplicate Address Detection with Hash Function

**DOI:** 10.1371/journal.pone.0151612

**Published:** 2016-03-18

**Authors:** GuangJia Song, ZhenZhou Ji

**Affiliations:** School of Computer Science and Technology, Harbin Institute of Technology, Harbin, China; Semmelweis University, HUNGARY

## Abstract

Duplicate address detection (DAD) is an important component of the address resolution protocol (ARP) and the neighbor discovery protocol (NDP). DAD determines whether an IP address is in conflict with other nodes. In traditional DAD, the target address to be detected is broadcast through the network, which provides convenience for malicious nodes to attack. A malicious node can send a spoofing reply to prevent the address configuration of a normal node, and thus, a denial-of-service attack is launched. This study proposes a hash method to hide the target address in DAD, which prevents an attack node from launching destination attacks. If the address of a normal node is identical to the detection address, then its hash value should be the same as the “Hash_64” field in the neighboring solicitation message. Consequently, DAD can be successfully completed. This process is called DAD-h. Simulation results indicate that address configuration using DAD-h has a considerably higher success rate when under attack compared with traditional DAD. Comparative analysis shows that DAD-h does not require third-party devices and considerable computing resources; it also provides a lightweight security resolution.

## Introduction

One of the main functions of a computer network is the exchange of data between nodes. In this process, packets are transferred from the source nodes to the destination nodes through various layers of intermediate devices, such as routers or switches. Packets can be delivered either directly or indirectly. The former occurs when both the source and destination nodes are on the same link or in the same local area network (LAN). In this case, the switch uses its own <Port, MAC> mapping table to locate the corresponding port of the destination media access control (MAC) address. The internet protocol (IP) packets are then forwarded directly to the port of the destination node. If the source and destination nodes are on different links, then the packets will be delivered indirectly. Packets must be sent through routers until they reach the network in which the destination node is located, and the local switch (or router) proceeds with a direct delivery [1]. Regardless of the delivery mode, knowing only the IP address is insufficient to deliver a packet; the MAC that corresponds to the IP address should also be identified. The process of obtaining a MAC address according to its IP address is called address resolution (i.e., neighbor discovery in IPv6) and is realized via the address resolution protocol (ARP) and the neighbor discovery protocol (NDP) [2][3].

In IPv4, ARP is used to complete address resolution. In internet protocol version 6 (IPv6), NDP is used for the same purpose. NDP is an important basic protocol for IPv6; it combines various enhanced IPv4 protocols, including ARP, the internet control message protocol (ICMP) routing discovery, and ICMP routing redirection. As the basic protocol for IPv6, NDP performs other functions, such as prefix discovery, neighbor unreachability detection (NUD), duplicate address detection (DAD), and stateless address autoconfiguration (SLAAC). For security reasons, the internet engineering task force (IETF) proposed the secure neighbor discovery (SEND) to enhance the security of NDP [4]. In terms of framework, both ARP and NDP minimally contains the following main parts:

1. address resolution,
2. duplicate address detection, and
3. cache maintenance.

As an important part of address resolution protocols (ARPs), DAD (in this study, DAD refers to all duplicate address detection processes in ARPs and differs from “DAD” in NDP) is vulnerable to attacks. The reason for its vulnerability is that traditional DAD assumes that all network nodes are credible; however, malicious nodes are ubiquitous in reality. When a normal node conducts DAD (assuming the target address is IP_*X*_; IP_*X*_ represents the target address of DAD in the remaining sections of this paper), malicious nodes have two ways to attack:

Method 1: By sending a forged reply, claiming that IP_*X*_ is in conflict;
Method 2: By launching DAD, with IP_*X*_ as its target address.

According to current protocols, both methods can lead to address configuration failure. Once address configuration fails, a node needs to configure other IP addresses and restart the DAD process. If the attack is continuous, the normal node will not be able to configure a new IP address nor access the network, thus launching a denial-of-service (DoS) attack.

To prevent a DoS attack, we proposed a new DAD process called DAD-h. This process uses the hash function to hide the target address of DAD, which prevents the attack node from identifying the address that will be used by the host, and thus, averts DoS attacks. The remaining sections of this paper are organized as follows. Section 2 presents the development of and related works on DAD. Section 3 introduces the algorithm and workflow of DAD-h. Section 4 provides a comparison between DAD and DAD-h, as well as between DAD-h and other typical security schemes. Section 5 provides a summary of the paper.

## Development of DAD and Related Works

### Development of DAD

**Fig 1:**
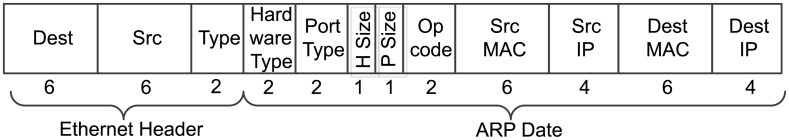
Packet format of ARP.

The basic format of ARP packets is shown in Fig 1. The MAC and IP addresses of the source node are indicated as “Src MAC” and “Src IP”, respectively, whereas those of the destination node are indicated as “Dest MAC” and “Dest IP”, respectively.

**Fig 2:**
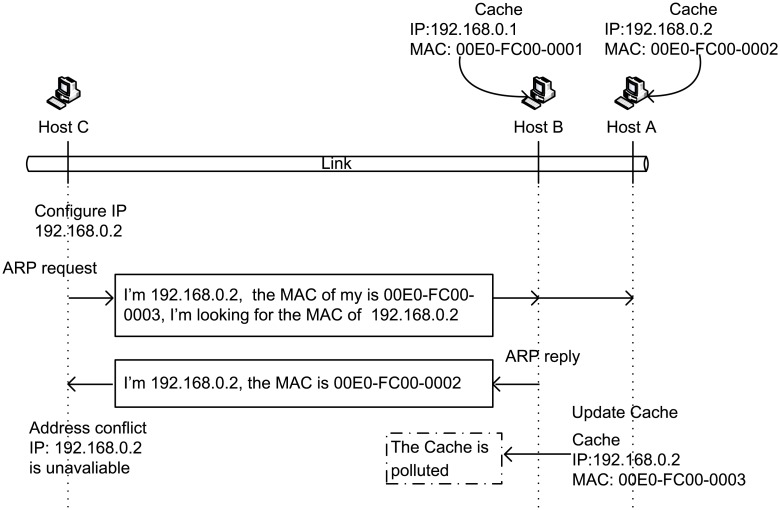
Cache pollution procedure.

ARP initially depends on gratuitous ARP for DAD. The gratuitous ARP process can be described as follows. When host A decides to use IP_*X*_ as its address, it must broadcast an ARP request to ensure that IP_*X*_ is not in conflict with other hosts. This ARP request differs from general ARP requests; that is, both of its “Src IP” and “Dest IP” fields are filled with IP_*X*_, and it aims to verify whether IP_*X*_ has already been used by another host. If host A receives a response to the broadcast, then IP_*X*_ is in conflict with another host. However, such detection process may cause cache pollution to other hosts. When a host receives a broadcast ARP request, it will update its cache according to the “Src IP” and “Src MAC” fields in the request because ARP has a mechanism for passively obtaining <IP, MAC> mapping. In this study, we present an example to demonstrate how failed address configuration pollutes the cache of other hosts, as shown in Fig 2.

Suppose three hosts exist in a LAN: hosts A, B, and C. The IP addresses of hosts A and B are 192.168.0.1 and 192.168.0.2, respectively, and their MAC addresses are 00E0-FC00-0001 and 00E0-FC00-0002, respectively. The initial states of host A’s cache and host B’s cache are shown at the top right corner of Fig 2. Host C is a joining host, and its MAC address is 00E0-FC00-0003. To conduct network communication, host C needs to configure an IP address. Assuming that host C configured 192.168.0.2 as its IP address, then host C broadcasts an ARP request for DAD. This request can be received by both hosts A and B. If host B determines that the destination address of the ARP request is the same as its IP address, then host B will send an ARP reply. When host C receives the response from host B, it realizes the conflict in address and must reconfigure the address, assuming that host C has finally configured 192.168.0.3 as its address. Table 1 presents the address information of the three hosts in this scenario.

**Table 1:** Address configuration information of hosts A, B, and C.

Host	IP	MAC
host A	192.168.0.1	00E0-FC00-0001
host B	192.168.0.2	00E0-FC00-0002
host C	192.168.0.3	00E0-FC00-0003

If the ARP request is received from host C, and host A determines that host C has a different target, then host A will not send an ARP reply. However, host A will update its cache according to the address information in the ARP request. The MAC address that corresponds to IP: 192.168.0.2 will be updated to 00E0-FC00-0003. By comparing the data presented in Table 1, we determine that the aforementioned address is an incorrect entry; that is, the cache of host A has been contaminated (see lower right corner of Fig 2). In the subsequent communication process, if host A wants to communicate with 192.168.0.2 (host B), then messages will be sent to 00E0-FC00-0003; and thus, the messages intended for host B will be received by host C instead.

To avoid cache pollution, RFC5227 proposed a new DAD method called address conflict detection (ACD) [5]. Two new packets are added in this method, namely, an ARP probe and an ARP announcement. An ARP probe is similar to an ARP request; however, its “Src IP” field is filled with “0.0.0.0” to reduce cache pollution. The ACD process is described as follows. If host A wants to use IP_*X*_, then host A has to first broadcast an ARP probe to confirm whether a conflict exists. If a conflict does not exist, then host A will send an ARP announcement, usually thrice. In the ARP announcement, the “Src IP” and “Dest IP” fields will be filled with the new address (IP_*X*_) and host A will announce that it will use IP_*X*_.

**Fig 3:**
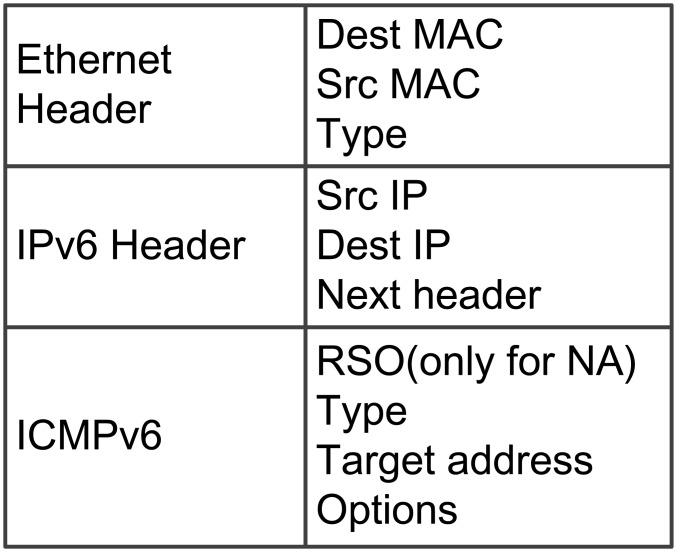
Format of an NDP message.

In NDP, detection mainly depends on neighbor solicitation (NS) and neighbor advertisement (NA). The format of an NDP message is shown in Fig 3. The “Target address” field typically stores the target address to be detected (or to be resolved). The “Options” field varies depending on the “Type” field of the message; it usually stores the MAC address of a node. The “Type” field represents the message type. The “Type” of NS is 135, whereas the “Type” of NA is 136. The “RSO” field exists only in NA.

**Fig 4:**
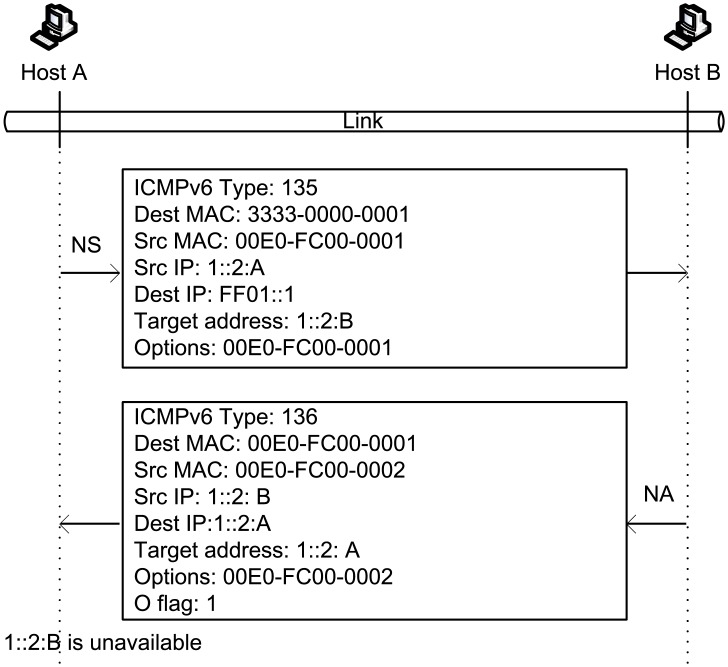
An example of DAD in NDP.

We present an example to illustrate the DAD process in NDP. Assume that the IPv6 address of host B is 1:: 2:B. If host A also wants to use 1:: 2:B as its address, then host A needs to broadcast an NS message to ensure that the new address is unique in LAN. After receiving the NS message, host B replies with an NA message to indicate that the address is in conflict. Examples of NS and NA messages are shown in Fig 4.

### Related works

At present, DAD faces three urgent problems: 1. Time delay; 2. overhead, and 3. security. This study focuses on the third problem.

#### Time-delay

In accordance with the current address resolution protocols, DAD is completed within 1–3 seconds. For some applications with high real-time requirements, such a delay is intolerable, particularly in mobile IPv6 (MIPv6). To reduce time delay, different improvement schemes according to the network environment have been proposed. For example, to achieve fast handover in MIPv6 environment, using a new IP address before completing DAD is recommended, or pro-active detection is used [6–8].

#### Overhead

In a wireless environment, such as mobile ad hoc networks and wireless sensor networks, DAD is launched when a node obtains a new IP address or during network separation and integration to avoid address conflict. However, node energy and computing resources are considerably important in a wireless environment. If DAD overhead is too large, then the survival of the network will be affected. To reduce overhead, a special address configuration or a special node to store node address information is used. By comparing with these special nodes, DAD can be completed, and thus, the consumption of network resources caused by flooding is prevented [9][10].

#### Security problem

In Section 1, we have mentioned that a DoS attack is the main security threat faced by DAD. In DAD, the target address of detection is public; thus, malicious nodes can send forged replies to fake an address conflict. A DoS attack does not only exist in IPv4 but also in IPv6, and with a greater risk. Two reasons are cited for this phenomenon.

1. Compared with that in IPv4, DAD occurs more frequently in IPv6. In the IPv4 environment, a host generally has only one IP address, and the address is very rarely changed. By contrast, DAD occurs more frequently in IPv6 because of the following reasons:
  - Multihoming of IPv6. IPv6 allows the host to have multiple types of addresses. The node can configure its address according to the network prefix in router advertisements to connect to different networks.
  - Deployment of MIPv6. In MIPv6, every time the mobile node enters a new network, the node has to configure a new network address to maintain communication with the outside; thus, DAD is launched.
  - Privacy protection. To prevent leakage of node privacy, the IETF proposed using a random interface identifier (IID), which should be changed regularly. This situation requires frequent replacement of the IPv6 address of the node [11][12].
2. SLAAC. As the main characteristic of NDP, SLAAC is the most common address allocation method for IPv6. It allows nodes to configure network address automatically without manual operation [13]. This feature facilitates address configuration, but also makes NDP vulnerable to DAD attacks. Manual operation can rapidly solve the problems encountered in DAD; thus, the administrator can also force a node to use a specific IP address. In SLAAC, however, one attack can lead to the failure of a large number of nodes to obtain an available address, such as using THC-IPv6 [14].

Few studies have examined DAD security. In [15], an integrated framework to prevent DAD attack was proposed. This framework should deploy a security server in LAN to run centralized management software. The server requires a static IP address and must ensure server safety. Management software needs to monitor all network traffic. All hosts have to exchange information with the server periodically to ensure that the server has the latest address information of LAN hosts. When a host conducts DAD, the security server should determine whether conflicts exist and then send a reply. The host will ignore DAD replies that do not come from the security server. Given that the security server which requires switch support to monitor network traffic is introduced, a single point of failure exists. Consequently, deployment cost is high.

NDPmon, a software program in Linux, is used to monitor NDP messages [16]. NDPmon should deploy a center server in LAN and is used by the host to monitor NDP messages. The neighbor discovery behavior of the host is used to determine whether an attack has occurred. If the MAC address in the “Options” field is inconsistent with the source MAC address in the Ethernet header, then the message will be considered an attack. Subsequently, NDPmon will send its system log to the center server and send an e-mail to the administrator. As a passive defense method, NDPmon only detects attacks but does not prevent them. NDPmon issues a warning for several normal network behaviors.

RFC 4861 proposed that Internet protocol security (IPsec) can be used to protect neighbor discovery under certain conditions; however, some difficulties in using IPSec to protect DAD are still encountered. The two sides completing key exchange is the premise that IPSec has played a role [17]; however, such a premise does not exist in DAD. IPSec protects point-to-point communication; however, DAD mainly depends on multicast communication, and the process occurs before point-to-point communication is established in most cases. Thus, IPSec cannot be used to protect DAD. Even if the IPSec mechanism plays a role between hosts A and C, host C can still use a forged reply to attack because the target address of detection is public.

To improve the security of NDP, the IETF proposed SEND as a solution. The main feature of SEND is a cryptographically generated address (CGA). CGA allows a node to prove that it has a particular address [18]. In the SEND environment, if host A performs DAD, then host C replies to claim that IP_*X*_ is in conflict, and host C should provide the original auxiliary parameters of CGA to prove that it possesses IP_*X*_. The characteristic of CGA is that the original auxiliary parameters cannot be inferred from CGA itself, which effectively prevents address spoofing. The disadvantage of SEND is that CGA generation requires considerable computation. When “Sec” parameter is increase by one, the calculation amount of CGA will increase 2^16^ times. This effect hampers SEND deployment. In [19], the use of a time-stopping algorithm in CGA generation was proposed according to the upper bound of running time to obtain an appropriate “Sec” value. The purpose of this procedure is to limit the time used for generating CGA and to ensure that CGA is generated within the specified time. In [20], a parallel computing algorithm was presented to shorten the computation time of CGA. In [21], the ECC (Elliptic Curves Cryptography) key was used to replace the RSA (Ron Rivest,Adi Shamir,Len Adleman) key to reduce computation time while achieving the same level of security. The ECC key is shorter, and the generated NDP messages are smaller. In addition to computational complexity, SEND messages increase signature, time-stamp, nonce, and other options [22]. Consequently, the SEND message is larger than the original NDP message, which increases communication overhead.

Source address validation implementation (SAVI) is a security mechanism that filters a packet according to the source address to prevent attacks; it aims to prevent attacks from the source. In the SAVI environment, the switch can bind an IP address to a switch Port. If the host sends a message that the source IP address is inconsistent with the binding information, then the switch will refuse to forward [23]. In [24], MAC was recommended to be used as one of the binding anchors. Deploying SAVI in LAN can prevent most attacks against NDP [25]. However, binding information in SAVI is extracted from the DAD message by monitoring network traffic. Therefore, SAVI does not check the NS message during DAD. Consequently, malicious nodes can use method 2 (mentioned in Section 1) to launch DoS attacks. SAVI requires a network device support; however, given that various network equipment manufacturers achieve simple network management protocol (SNMP) in different ways, deploying SAVI remains difficult.

## DAD-h

### Hash function

The hash function *h* has important applications in computer science and cryptography. It is mapped as *h*: {0,1}* → {0,1}^*n*^, where *h*: {0,1}* denotes a set of bit strings of any length, and {0,1}^*n*^ denotes a set of *n* bit strings [26]. On the basis of this definition, the hash function *h* can map a message *x* of any length to a short *y* with a fixed length. That is, *y* = *h*(*x*), where *x* is typically known as the pre-image, and y is typically called the message digest. Common hash functions include the message digest algorithm 5 (MD5) and the security hash algorithm 1 (SHA-1). A hash function is considered safe if the following three properties are achieved.

1. Resistance to a pre-image attack (one-way). For any given output *y*, finding an *x*, which makes *h*(*x*) = *y*, is computationally infeasible.
2. Resistance to a second pre-image attack. For any given input *x*, finding an input *x*′ that is unequal to *x*, which makes *h*(*x*) = *h*(*x*′), is computationally infeasible.
3. Resistance to a collision attack: Finding two unequal inputs *x* and *x*′, such that *h*(*x*) = *h*(*x*′), is computationally infeasible.

### DAD-h

#### Design goals of DAD-h

From the DAD process, the disclosure of key information (target address of detection) leads to the inherent vulnerability of DAD, and such disclosure allows malicious nodes to launch targeted attacks [27]. Hence, if the target address of detection can be hidden, then DoS attacks can be effectively prevented. In addition to achieving the basic function of duplicate address detection, the design goals of DAD-h are the following:

1. It does not leak the target address of DAD.
2. It can prevent DoS attack.

We uses the one-way characteristic of hash function to hide “Target address” field of DAD message; only the hash value of the “Target address” is public. Moreover, only the host with the specific IP address is allowed to know the real target address of DAD; other hosts only know the hash value of target address, so goals 1 and 2 are achieved.

#### Message format of DAD-h

**Fig 5:**
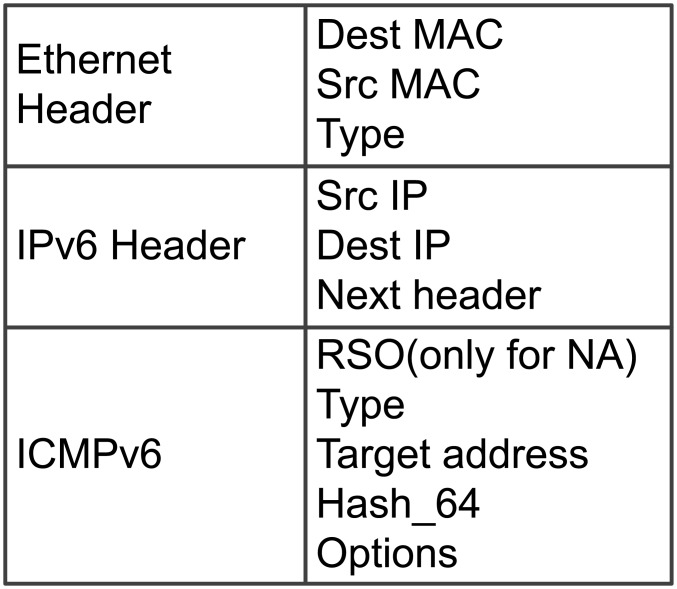
Message format of DAD-h.

**Fig 6:**
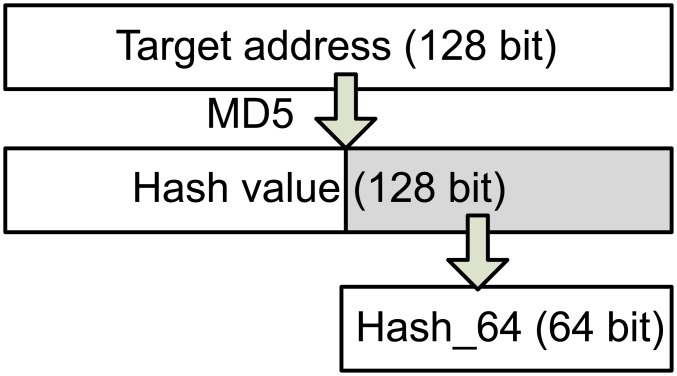
Calculation of the Hash_64 field.

The message format of DAD-h is illustrated in Fig 5. DAD-h uses two new message types, namely, NS_*DAD*−*h*_ and NA_*DAD*−*h*_, and its “Type” fields are 200 and 201, respectively. Compared with the NDP message, DAD-h adds a new field “Hash_64”, which stores the last 64 bits of the hash value of the “Target address” field of DAD. The calculation method of the “Hash_64” field is shown in Fig 6. If host A wants to use IP_*X*_ as its new address, then host A is required to calculate MD5 for IP_*X*_ before DAD is conducted. Subsequently, it intercepts the last 64 bits of the MD5 value to write it into the “Hash_64” field. This process is defined as a function H64 (IPv6_*address*).

#### Workflow of DAD-h

**Fig 7:**
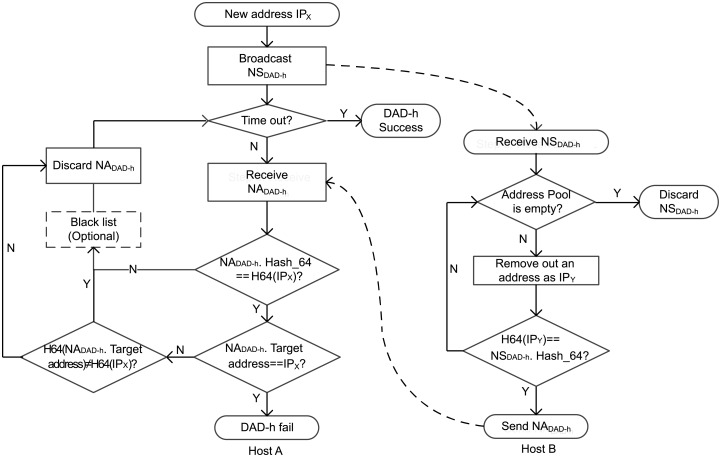
Flowchart of DAD-h.

The workflow of DAD-h is shown in Fig 7. In the following decription, we use IP_*A*_ and MAC_*A*_ represent the IP address and MAC address of host A, respectively, use IP_*B*_ and MAC_*B*_ represent the IP address and MAC address of host B, respectively.

When host A decides to use a new address IP_*X*_, it initially broadcasts an NS_*DAD*−*h*_. The detail of NS_*DAD*−*h*_ is shown in Table 2. Within a specified time, host A receives all NA_*DAD*−*h*_ and verify them. If an NA_*DAD*−*h*_ passed verification, then DAD-h fails and IP_*X*_ becomes unavailable. The algorithm used in this process is shown in Alg. 1.

**Algorithm 1:** send NS_*DAD*−*h*_ and receive NA_*DAD*−*h*_

1: **Input**: Internet Protocol address IP_*X*_

2: **Output**: true: DAD-h success; false: DAD-h fail

3: broadcast NS_*DAD*−*h*_

4: **while** DAD-h timeout ≠ true **do**

5:  receive NA_*DAD*−*h*_

6:  **if** NA_*DAD*−*h*._Hash_64 == H64(IP_*X*_) **then**

7:   **if** NA_*DAD*−*h*._Target address == IP_*X*_ **then**

8:    **return** false

9:   **else**

10:    **if** H64(NA_*DAD*−*h*._Target address) ≠H64(IP_*X*_)**then**

11:     add NA_*DAD*−*h*_.Src MAC into blacklist

12:    **end if**

13:    discard NA_*DAD*−*h*_

14:   **end if**

15:  **else**

16:   add NA_*DAD*−*h*_.Src MAC into blacklist

17:   discard NA_*DAD*−*h*_

18:  **end if**

19: **end while**

20: **return** true

**Table 2:** NS_*DAD*−*h*_.

Ethernet header	Dest MAC	3333-0000-0001
	Src MAC	MAC_*A*_
	Type	0x0806
IPv6 header	Src IP	IP_*A*_
	Dest IP	FF02::1
	Next header	0x3a
ICMPv6	Type	200
	Target address	::
	Hash_64	H64(IP_*X*_)
	Options	MAC_*A*_

When the other host (represented by host B) receives the NS_*DAD*−*h*_, it will search in its address pool to find an IP address (IP_*Y*_) that satisifies the equation:

$${\text{H64(IP}}_{Y}{\text{) = NS}}_{{}_{DAD-h}}.\text{Hash}\_64$$

The existence of IP_*Y*_ indicates a conflicting address. Host B then needs to send an NA_*DAD*−*h*_ as reply to host A. The algorithm used in this process is shown in Alg. 2. The detail of NA_*DAD*−*h*_ is shown in Table 3.

**Algorithm 2:** receive and verify NS_*DAD*−*h*_

1: **Input**: NS_*DAD*−*h*_

2: **Output**: true: send NA_*DAD*−*h*_ to reply; false: discard NS_*DAD*−*h*_

3: receive NS_*DAD*−*h*_

4: **while** address pool is not empty **do**

5:  remove out an IP address as IP_*Y*_

6:  **if** H64(IP_*Y*_)==NS_*DAD*−*h*._Hash_64 **then**

7:   send out NA_*DAD*−*h*_

8:   **return** true

9:  **end if**

10: **end while**

11: discard NS_*DAD*−*h*_

12: **return** false

**Table 3:** NA_*DAD*−*h*_.

Ethernet header	Dest MAC	MAC_*A*_
	Src MAC	MAC_*B*_
	Type	0x0806
IPv6 header	Src IP	IP_*B*_
	Dest IP	IP_*A*_
	Next header	0x3a
ICMPv6	RSO	O = 1
	Type	201
	Target address	IP_*Y*_
	Hash_64	H64(IP_*Y*_)
	Options	MAC_*B*_

In Alg. 1, the “blacklist” is an optional mechanism, it is based on the following three principles.

Principle 1: The “Hash_64” field value in NS_*DAD*−*h*_ is known; hence, if the “Hash_64” in NA_*DAD*−*h*_ does not match that in NS_*DAD*−*h*_, then the node should be considered malicious, and its MAC address should be added into the blacklist.
Principle 2: If the “Hash_64” field in NA_*DAD*−*h*_ is consistent with that in NS_*DAD*−*h*_ but the “Target address” field is not identical to IP_*X*_ and H64 (*Targetaddress*) ≠ Hash_64 field, then NA_*DAD*−*h*_ is considered a spoofing attack. Hence, the MAC address of the node should be added into the blacklist.
Principle 3: If SLAAC is used in the address configuration, and the IID is generated according to EUI-64 rules, then no address conflict should be evident in theory. If an address conflict occurs, then the MAC address of the reply node should be added into the blacklist to ensure the success rate of the secondary address configuration.

### Security analysis

#### Field length of “Hash_64” field

Suppose *n* nodes are present in LAN, wherein each node has *m* IPv6 addresses, the length of “Hash_64” field is *L*, then the hash collision probability in DAD-h process is:

$$\begin{array}{c} 1-\prod_{i=1}^{m\times n}\left(1-\frac{i}{2^{L}}\right) \end{array}$$ (1)

Proof:

First, we assume that the hash function is perfect, so the hash value is random and non-repetitive.

Given that *n* nodes are present in LAN, each node has *m* addresses. Thus, the total number of addresses in LAN is *m* × *n*, which means that *m* × *n* hash random values exist. Suppose the probability that these hash values do not collide with the “Hash_64” is *P*. Then,

$$\begin{array}{l} P=(\frac{2^{L}-1}{2^{L}})\times (\frac{2^{L}-2}{2^{L}})\times \cdots \times (\frac{2^{L}-m\times n+1}{2^{L}})\\ =(1-\frac{1}{2^{L}})\times (1-\frac{2}{2^{L}})\times \cdots \times (1-\frac{m\times n-1}{2^{L}})\\ =\prod_{i=1}^{m\times n}\left(1-\frac{i}{2^{L}}\right) \end{array}$$

Thus, the probability of collision is

$$\begin{array}{c} 1-\prod_{i=1}^{m\times n}\left(1-\frac{i}{2^{L}}\right) \end{array}$$

Proof is complete.

Thus, *L* is an important value; it determines how many reply messages will be generated in LAN. A shorter *L* leads to a higher security but also indicates more replies and bigger disturbance in LAN. By contrast, a longer *L* increases the possibility of being attacked, but introduces less reply messages. In this case study, *L* is set to 64. In the DAD-h, if 2^8^ nodes are present, with each node having 2^10^ IPv6 addresses, then the number of reply messages is

$$\begin{array}{c} 1-\prod_{i=1}^{2^{10}\times 2^{8}}\left(1-\frac{i}{2^{64}}\right)\approx 1-e^{\frac{-1}{2^{29}}} \end{array}$$ (2)

Which can be neglected.

#### Security of the “Hash_64” field

Assume that the network bandwidth is *M*, the length of “Hash_64” field is *L*, and the time of DAD-h process is *t*, then success rate of collision attack is no more than:

$$\begin{array}{c} 1-\prod_{i=1}^{\frac{M\times t}{90}}\left(1-\frac{i}{2^{L}}\right) \end{array}$$ (3)

Proof:

1. Assuming that the attack node has an unlimited computing ability means that the attacker can find all collision addresses within time *t*. The length of “Hash_64” field is *L*. Thus, there are 2^128−*L*^ collision addresses.
2. In IPv6, the message size of NDP is 90 K. The bandwidth is *M*. Thus, the number of NDP message that can be sent out in time *t* is:
  $$\begin{array}{c} n=\frac{M}{90}\times t \end{array}$$

That is, within time *t*, the attacker can send *n* collision addresses to attack at most. The probability *P* that the collision address is the same with the pre-address (IP_*X*_) is

$$\begin{array}{c} P=1-\prod_{i=1}^{m\times n}\left(1-\frac{i}{2^{L}}\right)=1-\prod_{i=1}^{\frac{M\times t}{90}}\left(1-\frac{i}{2^{L}}\right) \end{array}$$

Proof is complete.

Thus, if the network bandwidth is 10 G byte, the length of “Hash_64” field is 64 and DAD time is 3 s, then there are 2^64^ collision addresses, and the number *n* of NDP message that can be sent out in 3 s is

$$\begin{array}{c} n=\frac{10\times 2^{10}\times 2^{10}\times 3}{90} \end{array}$$

The success rate of the collision attack is:

$$\begin{array}{c} P=1-\prod_{i=1}^{n}\left(1-\frac{i}{2^{64}}\right)\approx 1-e^{\frac{-1}{2^{28}}} \end{array}$$

Thus, the success rate of the collision attack can be neglected in DAD-h.

### Example of DAD-h

We present an example to demonstrate the DAD-h process. The assumptions are that three hosts, namely, A, B, and C, are present in the network, and their address configuration information is as shown in Table 4.

**Table 4:** Address information of hosts A, B, and C.

Host	IP	MAC	Hash of IP
host A	1::2:A	0800-270c-0001	1d501fb0fe53ee99bbab3ef4685f2001
host B	1::2:B	0800-270c-0002	8ef841bd7e18a75e47941fa979a4bbad
host C	1::2:C	0800-270c-0002	20a6d4738c32a5f8b88d17760be9acd5

Assume that host A generates a new address 1::2:B. To determine whether the address is occupied, host A has to send NS_*DAD*−*h*_ to perform DAD. Host A fills in the “Hash_64” field with the last 64 bits of the hash value “8ef841bd7e18a75e47941fa979a4bbad” from 1::2:B (i.e., “47941fa979a4bbad”) and fills in the “Target address” field with “::”, which is an empty address. Both hosts B and C will receive this NS_*DAD*−*h*_.

Host C removes an IP address 1::2:C from the address pool, and the calculated hash value is “20a6d4738c32a5f8b88d17760be9acd5,” with the last 64 bits “b88d17760be9acd5” being different from the “Hash_64” field of NS_*DAD*−*h*_, and no more address is found in the address pool. Thus, host C discards NS_*DAD*−*h*_. If host C wants to attack host A, then it should forge NA_*DAD*−*h*_ and fill in the “Target address” of the forged NA_*DAD*−*h*_ with a correct address (1:: 2:B). However, host C only knows the hash value of the correct address and cannot obtain the original address according to the hash value; thus, host C cannot launch DoS attacks.

**Fig 8:**
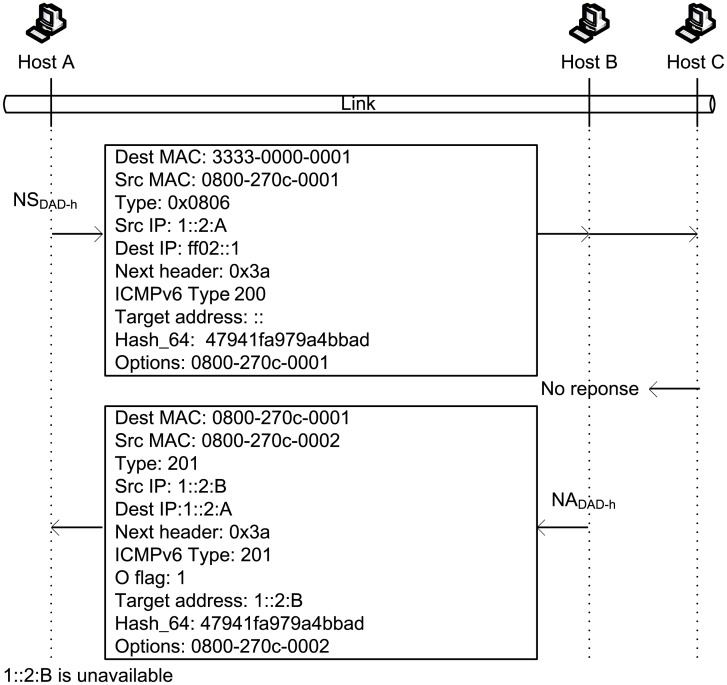
An example of DAD-h.

After host B receives NS_*DAD*−*h*_, it removes 1::2:B from its address pool and determines that the hash value of 1::2:B is “8ef841bd7e18a75e47941fa979a4bbad,” with its last 64 bits equal to the “Hash_64” field in NS_*DAD*−*h*_. Thus, node B replies with an NA_*DAD*−*h*_. Fig 8 illustrates the NS_*DAD*−*h*_ and NA_*DAD*−*h*_ used in this process. If host B has another address IP_*Z*_ with a hash value of “23cd002910efac1f47941fa979a4bbad” (i.e., the last 64 bits also match the “Hash_64” field), then node B must reply again with NA_*DAD*−*h*_.

When host A receives NA_*DAD*−*h*_, it determines that the “Hash_64” field is consistent with NS_*DAD*−*h*_. In addition, host A determines that “Target address” 1::2:B matches the target of DAD, and thus, host A realizes that an address conflict occurs.

By combining the workflow and the example, we can observe that the main differences between DAD-h and DAD are as follows:

1. DAD-h uses a new message format that adds a new field “Hash_64.” This format stores the hash value of the “Target address” field to ensure that the real target address of detection does not leak.
2. DAD-h adds a verification process. After host A receives the NA_*DAD*−*h*_, according to the “Hash_64” field and “Target address” field, the verification process can effectively filter out false replies.

## Experiment and Comparison

### Simulation experiment

At present, two widely used network simulation software packages are available: network simulation 2 (NS2) and optimized performance network engineering tool (OPNET). Compared with NS2, OPNET has a more friendly interface and supports more network protocols and equipment models. Thus, we choose OPNET as the experimental platform. The experimental machine consists of a personal computer with an AMD 5600 K CPU, 4 GB Corsair DDR3 memory, and Windows 7 Service Pack 3.

**Fig 9:**
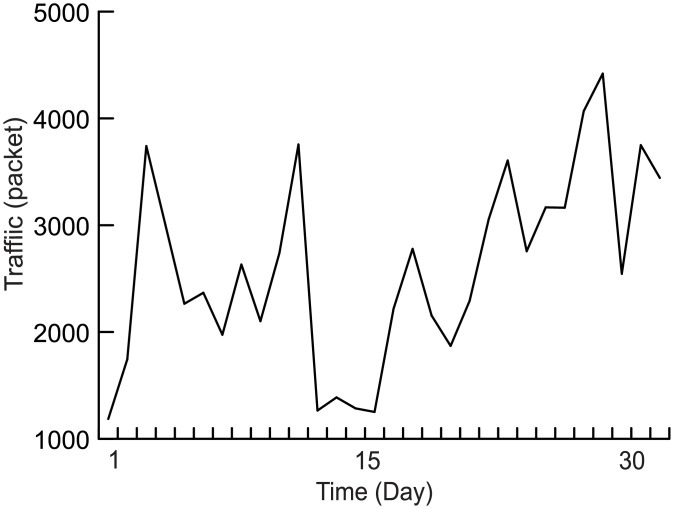
Average traffic in 30 days.

**Fig 10:**
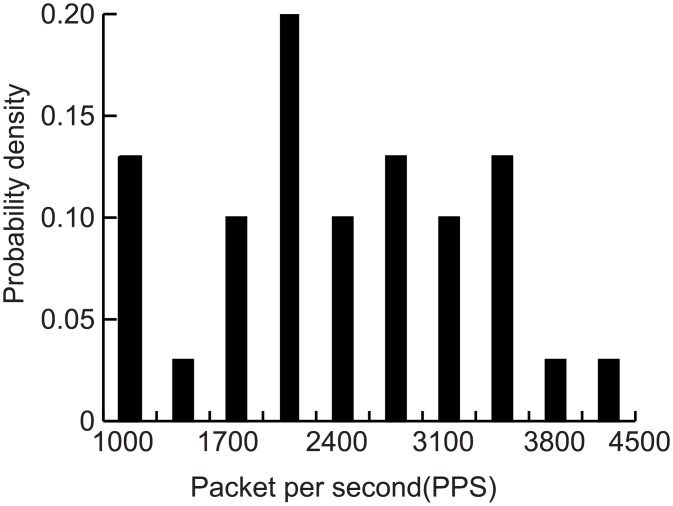
Data and probability distribution.

The network environment is LAN, which includes a switch node, an attack node, and seven normal nodes. A normal node includes two processors: Src1 and Src2. Src1 is used to generate background traffic. The distribution is sampled from the 30-day statistics of a university firewall (data acquisition tool: SolarWinds Orion; firewall model: Hillstone M6860, S1 Data). The data and distribution are shown in Figs 9 and 10, respectively. Src2 generates DAD messages with a uniform distribution and a mean of 1. Other experimental parameters are set as follows:

- Each node has 2^10^ addresses.
- The number of network prefix is 2^8^.
- Random address space is 2^32^.

The network node is less in order to simplify the experimental design and reduce the experimental error. The statistical error is 0.1 per thousand, which is equivalent to the link loss rate. Each node can have a large number of addresses, multiple network prefixes, and centralized random address space to increase the probability of address conflict.

The experiment statistic is address configuration success rate, it is defined as follows:

#### Address configuration success rate (ACSR)

When a host uses DAD process P to configure its address in the presence of attack. If a DAD process P is performed *n* times, and *m* times have failed, then the ACSR of P is:

$$\begin{array}{c} ACSR=1-\frac{m}{n} \end{array}$$ (4)

From definition of ACSR, we can conclude that if ACSR is 0, then attack is fully functional in P; if ACSR is 1, then P is immune to the attack. Thus, we can use the ACSR to measure a DAD process.

The experiments include two scenarios. Scenario 1 simulates DAD and DAD-h with the occurrence of an attack node. Attack node uses the following methods.

1. In DAD, the attack node forges an NA according to the “Target address” field of NS to respond.
2. In DAD-h, the attack node uses forged NA_*DAD*−*h*_ to respond, which “Target address” field is written a random IP address and the “Hash_64” is the same as that in NS_*DAD*−*h*_

**Fig 11:**
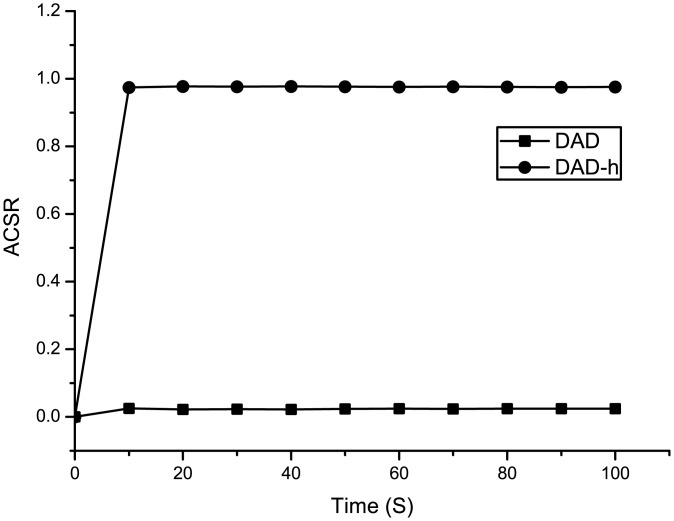
ACSR comparisons between DAD and DAD-h.

The experimental results are presented in Fig 11. The experiment results indicate that when the DAD is under attack, it has no ability to filter out the false DAD reply. The false reply causes the address configuration to fail. Thus, the ACSR of DAD is nearly zero (S2 Data). However, in DAD-h, the target address of DAD is not open. The attacker cannot figure out the real “Target address” field according to “Hash_64” field and “Target address” field. The probility that random address collision occurs with IP_*X*_ is extremely low (S3 Data). Thus, the ACSR of DAD-h is higher than DAD.

Scenario 2 simulates pseudo-collision attacks and SLAAC attacks against DAD-h.

#### Pseudo-collision attack

This method attempts to search for one or more collision addresses (the IP address with a hash value whose last 64 bits are the same as that in the “Hash_64” field) after the attack node receives NS_*DAD*−*h*_. Then, a number of NA_*DAD*−*h*_ is sent to increase the probability of a successful attack.

#### SLAAC attack

In SLAAC attack, the node can obtain an IP address by combining its own MAC address and network prefix according to “EUI-64.” Thus, the attack node can use the characteristics of SLAAC by combining the network prefix and source MAC address in the NS_*DAD*−*h*_ to infer the destination address of DAD.

**Fig 12:**
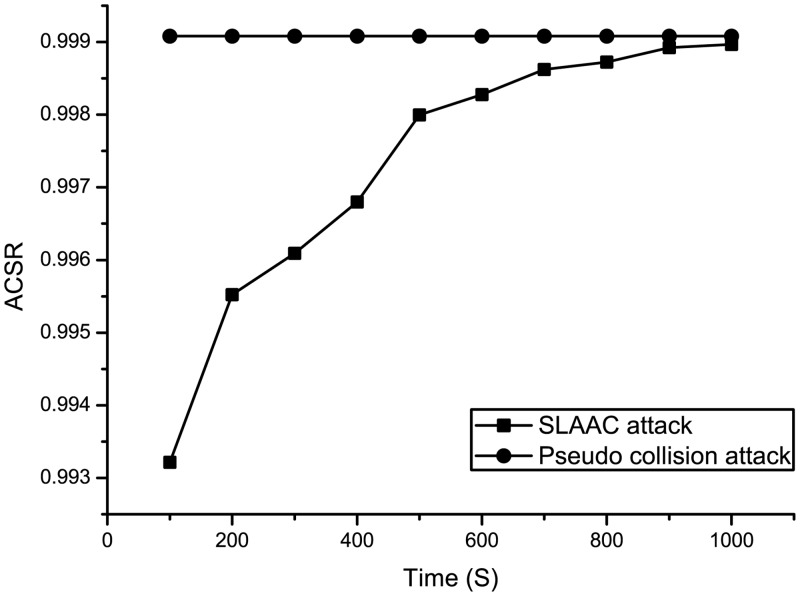
ACSR comparisons between Pseudo-collision attack and SLAAC attack.

In Scenario 2, DAD is set within 10 seconds (the normal time is 1–3 seconds). The experimental results are shown in Fig 12. For pseudo-collision attack, although the address space is 2^32^ and the attack node has 10 seconds to seek all collisions, host C remains incapable of locating the pre-image easily even under such generous conditions (S4 Data), as shown in Fig 12. Hence, the ACSR is significantly low. For a SLAAC attack, the address configuration is based on EUI-64. The attack node can use the method of combining the network prefix and MAC address to attack; thus, the success rate of address configuration is considerably low during the early stage of the experiment. Then, the blacklist mechanism will come into effect. It will record the MAC address of the attack node, and the subsequent attack packets will be discarded (S5 Data). In the second address configuration, node will use random IID; Thus, SLAAC attack does not work anymore, and the ACSR of the subsequent DAD-h process gradually increases and approaches to the ACSR of Pseudo-collision attack.

The aforementioned experimental results indicate that compared with DAD, DAD-h has two advantages:

1. The “Hash_64” field can effectively prevent attacks.
2. The blacklist mechanism can effectively prevent persistent attacks of the attack node.

### Comparative analysis

We compare DAD-h with several other typical mechanisms in the aspects of using cryptography, third-party devices, traffic monitoring, communication overhead, and database support. The compared results are presented in Table 5.

**Table 5:** Comparison of DAD-h with other security mechanisms.

	Cryptography used	Performance degradation	Third-party device	Traffic monitoring	Database support
SEND [4]	Yes	Moderate	Null	No	No
FCFS–SAVI [25]	No	Low	Switch/Router	Yes	Required
NDPmon [16]	No	Low	Secure server	Yes	Required
Rehman [15]	Yes	Moderate	Secure server	Yes	Required
DAD-h	No	Low	Null	No	No

When both parties use encrypted communication, the protocol performance deteriorates, as reflected in the method described in [4] and [15]. The methods used in [15, 16] are required to add an additional server in the network and must ensure server safety; however, this technique increases deployment cost. In [15], the security server itself requires periodic broadcasting to collect the <IP, MAC> mapping of all the hosts in LAN, which increases communication overhead. The method used in [15, 16, 25] requires a port mirror on the switch to monitor all network traffic to achieve message filtering. These methods require switch support and database support to record the corresponding <IP, MAC> relationship in the network. Compared with these solutions, DAD-h does not require monitoring the entire network traffic, and adding a third party in LAN and database support. In addition, the technique entails low deployment cost and provides a lightweight security mechanism.

## Conclusions

With an increasing number of network nodes and the extensive use of IPv6, DAD attacks pose a serious threat to network security. In traditional DAD, the host discloses the target address of DAD, which allows all network nodes to know the new address used by the host, and consequently, malicious nodes can forge replies to launch DoS attacks. DAD-h uses the one-way characteristic of the hash function to hide the target address during DAD; this technique only opens the hash value of the target address. The malicious node cannot forge a reply based on the “Target address” field. Simultaneously, DAD-h uses the blacklist mechanism to prevent the persistent attacks of malicious nodes. The simulation results show that DAD-h has a higher address configuration success rate than DAD under DoS attack. Compared with other security schemes, DAD-h exhibits advantages in terms of using network equipment, network traffic monitoring, and protocol performance.

## Supporting Information

S1 Data Average traffic in 30 days. (DAT)

S2 Data Address configuration success rate of DAD. (DAT)

S3 Data Address configuration success rate of DAD-h. (DAT)

S4 Data Address configuration success rate of Pseudo-collision attack. (DAT)

S5 Data Address configuration success rate of SLAAC attack. (DAT)
